# Associations of Baroreflex Sensitivity, Heart Rate Variability, and Initial Orthostatic Hypotension with Prenatal and Recent Postnatal Methylmercury Exposure in the Seychelles Child Development Study at Age 19 Years

**DOI:** 10.3390/ijerph120303395

**Published:** 2015-03-23

**Authors:** Daniel Périard, Bujar Beqiraj, Daniel Hayoz, Bharathi Viswanathan, Katie Evans, Sally W. Thurston, Philip W. Davidson, Gary J. Myers, Pascal Bovet

**Affiliations:** 1 Service d’Angiologie, Hôpital Cantonal, Fribourg 1700, Switzerland; E-Mails: beqirajbujar@gmail.com (B.B.); daniel.hayoz@h-fr.ch (D.H.); 2 Section of Non Communicable Diseases, Ministry of Health, Victoria, Seychelles; E-Mails: barathi.viswanathan@health.gov.sc (B.V.); pascal.bovet@chuv.ch (P.B.); 3 Department of Biostatistics, University of Rochester, Rochester, NY 14642, USA; E-Mails: katie_evans@urmc.rochester.edu (K.E.); sally_thurston@urmc.rochester.edu (S.W.T.); 4 Department of Pediatrics, University of Rochester, Rochester, NY 14642, USA; E-Mails: philip_davidson@urmc.rochester.edu (P.W.D.); gary_myers@urmc.rochester.edu (G.J.M.); 5 Institute of Social and Preventive Medicine, Lausanne University Hospital, CHUV, Lausanne 1010, Switzerland

**Keywords:** methyl mercury, mercury, baroreflex sensitivity, heart rate variability

## Abstract

*Background:* A few studies have suggested an association between prenatal exposure to methylmercury and decreased heart rate variability (HRV) related to autonomic heart function, but no study has examined this association using baroreflex sensitivity (BRS). In this study we assessed the distribution of BRS and immediate orthostatic hypotension (IOH) in young Seychellois adults and their associations with exposure to prenatal and recent postnatal methylmercury. *Methods:* Subjects in theSeychelles Child Development Study (SCDS) main cohort were evaluated at age 19 years. Non-invasive beat-to-beat blood pressure (BP) monitoring (Finapres, Ohmeda) was performed at rest and during active standing in 95 consecutive subjects. Recent postnatal mercury exposure was measured in subjects’ hair at the age of 19 years and prenatal exposure in maternal hair grown during pregnancy. BRS was estimated by sequence analysis to identify spontaneous ascending and descending BP ramps. HRV was estimated by the following markers: PNN50 (relative numbers of normal-to-normal intervals which are shorter by more than 50 ms than the immediately following normal-to-normal intervals); rMSSD (root mean of the squared sum of successive interval differences); LF/HF (low frequency/high frequency component ratio); ratio of the mean expiratory/inspiratory RR intervals (EI ratio); and the ratio between the longest RR interval 30 s after active standing and the shortest RR interval at 15 s (Max30/Min15). IOH was estimated by the deepest BP fall within the first 15 s after active standing up. *Results:* Prenatal MeHg exposures were similar in boys and girls (6.7 ± 4.3, 6.7 ± 3.8 ng/g) but recent postnatal mercury levels were higher in males than females (11.2 ± 5.8 vs 7.9 ± 4.3 ng/g, *p* = 0.003). Markers of autonomic heart rate control were within the normal range (BRS: 24.8 ± 7 ms/mm Hg, PNN50: 24.9 ± 6.8%, rMSSD: 68 ± 22, LF/HF: 0.61 ± 0.28) in both sexes. After standing, 51.4% of subjects had a transient systolic BP drop >40 mm Hg, but only 5.3% reported dizziness or had syncope. Prenatal and recent postnatal MeHg levels, overall, were not associated with BRS, E/I ratio, PNN50, rMSSD, LF/HF ratio, Max30/Min15 ratio, and IOH. *Conclusions:* This study provides no support for the hypothesis that prenatal or recent postnatal MeHg exposure from fish consumption is associated with impaired autonomic heart rate control.

## 1. Introduction

Prenatal and postnatal exposures to high levels of methyl mercury (MeHg) are associated with detrimental effects on health. Because MeHg accumulates in fish, studies on the toxicity of MeHg have often been carried out in populations with high ocean fish intake, e.g., the Seychelles, or in populations with high consumption of seafood including mammals, e.g., the Faeroes Islands, Japan, and Arctic Quebec [1]. In these studies, the primary focus has generally been on possible developmental effects of prenatal MeHg exposure, but a few studies also suggest a cardiovascular impact, including increased blood pressure (BP) [2,3,4,5] and decreased heart rate variability (HRV) [6,7,8,9]. However, the reported associations of mercury with cardiovascular outcomes have often been either weak or inconsistent across different studies [10].

To our knowledge, autonomic heart function in relation to MeHg exposure has been previously only evaluated through HRV estimation, a measure that requires simple three lead electrocardiogram long recordings. In contrast, a direct and more accurate estimation of the autonomic heart function can be made by measuring the baroreflex sensitivity (BRS). BRS requires continuous monitoring of BP and heart rate, e.g., continuous, non-invasive, beat-to-beat finger BP monitoring (e.g., Finapres, Ohmeda, BOC Health care, USA, which performs 200 BP measurements per second). BRS measures the active heart rate frequency response that immediately follows a variation in BP, e.g., BP variation along breath cycles in lying subjects. BRS is a very reliable marker of autonomic heart function and it can assess the integrity of the sympathetic and cardio-inhibitory nervous system. Impaired BRS has been shown to be associated with major cardiac outcomes and sudden death [11,12]. Reduced HRV has also been shown to be associated with increased mortality in persons with coronary heart diseases or diabetes [13,14]. No prior studies have assessed the association between MeHg and BRS in humans.

The Seychelles Child Development Study (SCDS) was designed to assess the impact of prenatal exposure to MeHg on developmental outcomes. At the age of 19 years, we examined the main cohort subjects for autonomic heart function in relation to prenatal and recent postnatal MeHg exposures from fish consumption. The current study has two aims. First it provides data on the distribution of BRS, HRV, and initial transient hypotension (IOT) in young healthy adults of the Seychelles. Such descriptive data is sparse in youth and particularly so in the population in the African region. Second it examines the association between prenatal and recent postnatal MeHg exposures and heart autonomic function.

## 2. Methods

### 2.1. Participants Selection

This study is based on data from a sub-study of the main cohort in the Seychelles Child Development Study (SCDS) led by the University of Rochester (New York, NY, USA) and funded by the NIH (USA). The SCDS aims are to assess the long-term effect of prenatal and recent postnatal exposures to MeHg from fish consumption on children’s neuro-cognitive development. The main cohort was enrolled in 1989–90 at the age of 6 months and has been evaluated regularly thereafter for developmental outcomes. The current study is based on results gathered during an evaluation when subjects were 19 years of age and took place between April 2008 and June 2009. Ninety-five consecutive subjects were evaluated with beat-to-beat blood pressure monitoring. The study was approved by the ethical committees of both the Ministry of Health of the Republic of Seychelles and the University of Rochester. All subjects were over 18 years of age and provided a written consent.

### 2.2. Physical Examination and Mercury Levels

Subjects were examined in the morning, after a 12-hour fast. Clinical measurements (weight, height, blood pressure) were performed using standard techniques. Prenatal MeHg concentration was available for all subjects and was held blinded by the University of Rochester. Prenatal MeHg exposure was measured as the average concentration of total Hg (THg) in the longest available segment of maternal hair growing during pregnancy. Recent postnatal MeHg exposure was measured as the average concentration of THg in the 1 cm of hair closest to the scalp, taken at the 19-year evaluation. Methods used for the measurement of mercury in hair in this study have been detailed elsewhere [15]. Of note, the unit for mercury in hair is usually expressed ppm or ng/g, with 1 ppm = 1 ng (of mercury) per g (of hair). Because over 80% of Hg in hair is MeHg [16] we refer to “MeHg” for mercury measurements in hair in this text.

### 2.3. Baroreflex Sensibility and Heart Rate Variability

Subjects were tested in a quiet room, lying on a bed, with their head tilted at 20 °C. They were asked to breathe slowly and regularly. Each recording was preceded by a 5 min resting pause. BP and heart rate (HR) were continuously monitored with a noninvasive finger cuff (Finapres, Ohmeda, BOC Health care, USA). BP and heart rate signals were digitalized at 200 Hz and stored for further analysis. Brachial BP was measured before each recording for calibration. The BRS was estimated by sequence analysis to identify spontaneous ascending and descending BP ramps [17,18]. HRV was estimated by the following four indexes based on RR interval analysis: the relative numbers of normal-to-normal intervals which are shorter by more than 50 ms than the immediately following normal-to-normal interval (PNN50), the root mean of the squared sum of successive interval differences (rMSSD), the low frequency/high frequency component ratio (LF/HF) of RR variability by spectral analysis after fast Fourier transformation [19,20], and the ratio of the mean expiratory/inspiratory RR intervals (E/I ratio) observed over a 30-second record at rest [21].

### 2.4. Initial Orthostatic Hypotension (IOH)

IOH describes an excessive BP fall within 15 seconds after active standing. It is a trigger of loss of consciousness in 3%–10% of both young and old subjects [22]. Only continuous beat-to-beat BP measurement during an active standing up maneuver can document this condition. The physiology of IOH relates to a temporary mismatch between cardiac output and vascular resistance. IOH is characterized by an initial heart rate increase (reflecting an abrupt inhibition of vagal activity upon standing), followed by a strong BP fall (reflecting a fall in systemic vascular resistance in response to increased blood flow to the right atrium when there is leg and abdominal muscle contraction upon standing), followed by a secondary rebound increase in BP after approximately 10 s (reflecting sympathetic vasoconstriction in the peripheral vasculature, likely through the baroreflex and induced by hypotension) [23]. Subjects were questioned about dizziness while standing up and investigators were aware of possible syncope. The lower systolic and diastolic BP values at nadir were compared to the BP before standing up, and the time duration to nadir was assessed. The ratio between the longest RR interval 30 s after standing and the shortest RR interval at 15 s (Max30/Min15) was used as another marker of HRV [21].

### 2.5. Statistical Analysis

Proportions were compared using Fisher’s exact test, and continuous variables were compared by the Student’s *t*-test. Statistical significance was considered for two sided *p*-values < 0.05. For each Finapres outcome, we examined the effects of prenatal and recent postnatal MeHg exposures in separate linear regression models. All models were adjusted for child’s sex, birth weight, and current BMI. Models were fit both with and then without a MeHg by sex interaction. Unless otherwise noted, results from the non-interaction model are reported. An additional model with recent postnatal MeHg also adjusted for activity index (sedentary, moderate, or active) and current postnatal omega 3 and omega 6 polyunsaturated fatty acids (PUFA). Model assumptions were checked using standard methods [24]. If regression assumptions were violated, the outcome was transformed and the assumptions re-checked. When conclusions were unaffected by the outcome transformation, results are presented from the untransformed model. Statistical analyses were performed using R version 3.0.2.

## 3. Results

### 3.1. Subjects’ Characteristics

Among the 779 subjects followed from birth to the current examination at age of 19 years, 95 (47 males) consecutive young adults (age 19.5 ± 0.31 years) were included in this sub-study. The majority of subjects were of African descent. A complete examination and signal recording was obtained for all included subjects. Clinical characteristics of the subjects are presented in Table 1. Mean ± SD systolic/diastolic blood pressure was 120/66 ± 10/8 mm Hg in males and 110/68 ± 10/8 mm Hg in females. Mean prenatal MeHg exposure concentrations were similar in both groups (6.7 ± 4.3, 6.7 ± 3.8 ng/g). Recent postnatal MeHg levels were higher in males compared to females (11.2 ± 5.8 *vs.* 7.9 ± 4.3 ng/g, *p* = 0.003).

### 3.2. Autonomic Heart Function

All markers of autonomic heart function were within the normal range (BRS: 25 ± 7 ms/mm Hg; PNN50: 24.9% ± 6.8%; rMSSD: 68 ± 22; LF/HF: 0.61 ± 0.28; E/I ratio: 1.44 ± 0.22). At active standing, five subjects (5.3%) had dizziness and one of these five (1.1%) had syncope. Systolic BP dropped on average by 47 mm Hg for males and 34 mm Hg for females (*p* = 0.002) after a mean delay of 11 seconds. Overall 51.4% had a systolic BP drop larger than 40 mm Hg (Table 2).

**Table 1:** Baseline characteristics of subjects.

Variables	Males					Females					
	*n*	Mean	SD	Min	Max	*n*	Mean	SD	Min	Max	*p* value
Age (years)	46	19.6	0.3	18.9	20.6	48	19.5	0.3	18.9	20.3	0.154
Height (cm)	47	174.9	7.7	158.0	192.0	48	161.9	5.4	152.3	176.0	<0.001
Weight (kg)	47	65.0	11.4	45.8	107.2	48	58.5	13.7	36.7	98.0	0.014
BMI (kg/m^2^)	47	21.3	3.7	14.9	35.5	48	22.3	5.0	14.5	39.7	0.260
Systolic BP (mm Hg)	47	120.5	9.9	101.0	143.3	48	110.4	10.4	89.3	156.3	<0.001
Diastolic BP (mm Hg)	47	66.1	7.5	51.3	87.7	48	68.3	8.0	51.7	102.3	0.175
Prenatal MeHg (maternal hair in ng/g)	47	6.7	4.3	0.7	20.0	48	6.7	3.8	1.7	21.3	0.980
Recent postnatal MeHg (subjects hair in ng/g)	41	11.2	5.8	2.9	28.1	45	7.9	4.3	2.0	20.5	0.003

### 3.3. Association with Methylmercury Exposure

There was a significant sex by MeHg interaction only for prenatal MeHg exposure and rMSSD (*p* = 0.023). The prenatal MeHg slope was significant only for males (male slope 1.99, *p* = 0.014; female slope −0.80, *p* = 0.378) and indicated improved HR variability. In main effects models, prenatal and recent postnatal MeHg exposures were not significantly associated with BRS, PNN50, rMSSD, LF/HF ratio, E/I ratio and Max30/Min15 ratio (Table 3). In models that additionally adjusted for activity level and postnatal omega 3 and omega 6 polyunsaturated fatty acids (PUFA), neither PUFA level was a significant predictor for any of the outcomes.

**Table 2:** Baroreflex sensitivity, heart rate variability and initial orthostatic hypotension in young healthy adults in the Seychelles.

Variables	Males			Females			*P*
	*n*	Mean or %	SD	*n*	Mean or %	SD	
BRS (msec/mm Hg)	41	25.4	6.7	40	24.2	7.5	0.451
E/I ratio	45	1.44	0.25	40	1.45	0.19	0.804
PNN50 ratio	40	0.26	0.07	42	0.24	0.07	0.366
rMSSD ratio	40	71.0	23.4	42	64.9	20.7	0.212
LF/HF ratio	38	0.61	0.27	41	0.61	0.30	0.937
Max30/Min15 ratio	34	1.17	0.23	38	1.22	0.22	0.419
Dizziness at standing (%)	47	4.0	48	6.0	0.740
Syncope at standing (%)	47	2.0	48	0.0	0.495
Time to BP nadir (s)	34	10.7	1.9	38	11.0	2.3	0.579
Systolic BP fall (mm Hg)	34	47.4	20.4	38	34.2	13.0	0.002
Diastolic BP fall (mm Hg)	34	23.5	14.5	38	21.9	11.7	0.605
Systolic BP fall >40 mm Hg (%)	34	71.0		38	34.0		0.002
Diastolic BP fall >25 mm Hg (%)	34	47.0		38	42.0		0.813
Heart rate at baseline (bpm)	34	67.3	11.5	38	78.6	13.6	<0.001
Heart rate at standing (bpm)	34	91.1	17.7	38	104.5	13.0	<0.001

BRS: baroreflex sensitivity; E/I ratio: ratio of the mean expiratory/inspiratory RR intervals; PNN50: relative numbers of normal-to-normal intervals which are shorter by more than 50 ms than the immediately following normal-to-normal intervals; rMSSD: root mean of the squared sum of successive interval differences; LF/HF ratio: low frequency/high frequency component ratio; Max30/Min15 ratio: ratio between the longest RR interval 30 s after standing and the shortest RR interval at 15 s; *P* refers to *p* value.

## 4. Discussion

In this study, we assessed the active autonomic regulation of the heart rate in response to spontaneous BP fluctuations at rest in the supine position and in response to a provoked BP drop (IOH at active standing). We found that the autonomic heart function response to spontaneous or provoked BP variations was preserved in this population of young adults of predominantly African descent [25]. Overall, there were no adverse associations of autonomic heart function parameters with prenatal or recent postnatal MeHg exposures in a population with an exposure from fish consumption significantly higher than is present in most developed countries [26]. 

To our knowledge, this study is the first to evaluate the potential autonomic association with prenatal or recent postnatal MeHg exposures using markers of the cardio-inhibitory baroreceptor system in the supine position and with an active orthostatic maneuver. A few previous studies have assessed the relation between mercury exposure and HRV measured by 24-h ECG monitoring. However, the variability of the RR intervals can be substantially affected by concomitant physical activity and other movements among active individuals during a 24-h recording period. In this study, we chose to rely on the gold standard for accurately assessing autonomic heart function through continuous beat-to-beat BP monitoring, and RR signals, in a tightly controlled environment.

**Table 3:** Covariate-adjusted associations between prenatal MeHg exposure (top half of table) and recent postnatal MeHg exposure (bottom half of table) and baroreflex sensitivity, heart rate variability and initial orthostatic hypotension in young healthy adults in the Seychelles. Results for each outcome are fit in separate models adjusting for either prenatal MeHg or recent postnatal MeHg exposure. For rMSSD and prenatal MeHg exposure only, there was a significant sex by prenatal MeHg interaction, for which the prenatal MeHg slope was positive (Coef: 1.99) and significant (*P* = 0.014) for males only.

Variables	BRS			E/I ratio			PNN50			rMSSD			LF/HF ratio			Max30/Min15 ratio		
	Coef	SE	*P*	Coef	SE	*P*	Coef	SE	*P*	Coef	SE	*P*	Coef	SE	*P*	Coef	SE	*P*
***Prenatal MeHg exposure***																		
Prenatal MeHg	0.176	0.192	0.362	0.004	0.006	0.548	0.001	0.002	0.551	0.784	0.613	0.205	0.006	0.008	0.486	−0.004	0.007	0.534
Birth weight	0.684	1.579	0.666	−0.012	0.049	0.806	−0.004	0.015	0.775	0.940	4.901	0.848	−0.062	0.065	0.340	−0.015	0.050	0.758
Current BMI	**−0.423**	**0.171**	**0.015**	−0.004	0.005	0.478	−0.001	0.002	0.422	−0.871	0.537	0.109	−0.003	0.007	0.683	0.001	0.007	0.873
Male (vs. female)	0.548	1.558	0.726	−0.016	0.049	0.744	0.012	0.015	0.424	5.120	4.869	0.296	−0.007	0.065	0.917	−0.041	0.054	0.449
*n*	81			85			82			82			79			72		
***Recent postnatal MeHg exposure***																		
Recent postnatal MeHg	−0.072	0.164	0.664	−0.002	0.005	0.687	0.001	0.002	0.686	0.136	0.506	0.789	−0.011	0.007	0.116	0.006	0.006	0.277
Birth weight	0.610	1.665	0.715	0.003	0.046	0.947	−0.004	0.016	0.819	2.047	5.052	0.687	−0.039	0.067	0.560	−0.003	0.053	0.956
Current BMI	**−0.447**	**0.180**	**0.015**	−0.003	0.005	0.558	−0.001	0.002	0.545	−0.693	0.557	0.218	−0.003	0.007	0.687	0.002	0.007	0.739
Male (*vs.* female)	0.726	1.815	0.690	−0.039	0.050	0.433	0.010	0.017	0.562	5.743	5.435	0.294	0.021	0.073	0.778	−0.053	0.057	0.355
*n*	73			77			76			76			73			68		

BRS: baroreflex sensitivity; E/I ratio: ratio of the mean expiratory/inspiratory RR intervals; PNN50: relative numbers of normal-to-normal intervals which are shorter by more than 50 ms than the immediately following normal-to-normal intervals; rMSSD: root mean of the squared sum of successive interval differences; LF/HF ratio: low frequency/high frequency component ratio; Max30/Min15 ratio: ratio between the longest RR interval 30 s after standing and the shortest RR interval at 15 s; *P* refers to *p* value.

Our study has a number of strengths. We used beat-to-beat blood pressure monitoring to provide highly specific measurements of autonomic heart function. The subjects studied had MeHg exposures significantly higher than in developed countries and a wide range of exposures including individuals with current postnatal values as high as 28.2 ng/g (median: 8.7; 90th percentile: 16.8). In addition, the young age of our subjects minimized the interference of other factors that might be associated with impaired autonomic heart function (e.g., diabetes or other age related degenerative conditions). Our study also has some limitations. The sample size was relatively small, thus, limiting our ability to exclude associations of small magnitude or adequately evaluate interactions. In addition, the recent postnatal MeHg exposure used in this study represents only exposure during a recent one-month period of time. We could not adjust our analyses to lead levels as lead was not measured when pre-natal exposure to MeHg was measured. Other studies done later showed fairly low levels of lead [16] and low levels of PCBs [27]. 

Given the strengths and limitations of this study, four lines of argument provide evidence against a strong association between mercury and cardiac autonomic dysfunction in this population. First, our study subjects had a relatively high average BRS (25 ms/mm Hg) when compared to reference values for adults or the few available studies on children and preadolescents. In a sample of healthy preadolescents aged 10 to 13 years in Europe, Dietrich and colleagues found mean BRS of 15.3 msec/mm Hg in the supine position [28]. In another study of 162 young subjects (11 to 21 years old) in Europe the mean BRS was approximately 10 ms/mm Hg for healthy subjects, 6 ms/mm Hg for patients with hypertension and 7 ms/mm Hg for patients with “pseudohypertension” (white coat effect and normal ambulatory BP) [29]. Although fish consumption and MeHg exposure were both high in Seychelles compared to other populations, BRS values were higher and thus more variable in these young subjects than among healthy subjects studied in the preceding two European studies [24,25]. Secondly, none of our 95 patients had a BRS below 10 msec/mm Hg, which is the cut off for abnormal values. Third, we found large variability in all five different HRV markers, even in the supine position at rest. Fourth, all subjects showed a marked initial orthostatic hypotension followed by a subsequent adequate correction, which is characteristic of a normal response to stimulation of the autonomic cardiac reflex, and we did not observe any attenuation of the BP fall or any delay to BP nadir that could have suggested an impairment of heart autonomic function through either or both cardio-inhibitory or cardio-stimulating innervations.

## 5. Conclusions

In conclusion, using highly specific cardiac autonomic parameters derived from beat-to-beat BP monitoring, we found normal autonomic heart function in a cohort of young Seychellois adults. 
